# Changes in Relationship Commitment Across the Transition to Parenthood: Pre-pregnancy Happiness as a Protective Resource

**DOI:** 10.3389/fpsyg.2021.622160

**Published:** 2021-02-16

**Authors:** Hagar Ter Kuile, Catrin Finkenauer, Tanja van der Lippe, Esther S. Kluwer

**Affiliations:** ^1^Department of Social, Health & Organisational Psychology, Utrecht University, Utrecht, Netherlands; ^2^Department of Interdisciplinary Social Science, Utrecht University, Utrecht, Netherlands; ^3^Department of Sociology, Utrecht University, Utrecht, Netherlands; ^4^Behavioral Science Institute, Radboud University Nijmegen, Nijmegen, Netherlands

**Keywords:** transition to parenthood, commitment, happiness, vulnerability-stress-adaptation model, actor-partner interdependence model, Mplus

## Abstract

The transition to parenthood is both a joyous and a challenging event in a relationship. Studies to date have found mostly negative effects of the birth of the first child on the parental relationship. We propose that partners' pre-pregnancy individual happiness may serve as a buffer against these negative effects. We predicted that parents who are happy prior to pregnancy fare better in terms of relationship commitment after childbirth than unhappy parents. To test our prediction, we used data of a 5-wave longitudinal study among 109 Dutch newlywed couples who had their first child during the study and a comparison group of 55 couples who remained childless. We found that the relationship commitment of fathers with higher pre-pregnancy happiness and fathers with a partner with higher pre-pregnancy happiness increased slightly in the years after childbirth, whereas the relationship commitment of fathers with lower pre-pregnancy happiness and fathers with a partner with lower pre-pregnancy happiness decreased. In addition, the relationship commitment of mothers with a happier partner prior to pregnancy decreased only slightly across the transition to parenthood but showed a steeper decline for mothers with a partner with average or lower pre-pregnancy happiness. In line with the idea that happiness acts as a resource when partners have to deal with relationship challenges, individual happiness predicted changes in relationship commitment for parents, but not for partners who remained childless.

## Introduction

The transition to parenthood is not only one of the most joyous life events but it can also be a challenging time in the relationship. Having the first child requires adaptation that can be accompanied by parental stress (Perren et al., 2005) and relational turbulence (Theiss et al., 2013). The general view that has dominated the literature is that the transition to parenthood has mostly negative effects on the parental relationship. Indeed, most studies show, on average, a small but reliable decrease in relationship functioning after child-birth (for reviews, see Twenge et al., 2003; Mitnick et al., 2009; Kluwer, 2010; Doss and Rhoades, 2017). Recently, however, it is recognized that there is important variability in how couples respond to the transition to parenthood (Doss and Rhoades, 2017). Some parents experience a decrease, while others experience no change or even an increase in relationship functioning (e.g., Holmes et al., 2013; Ter Kuile et al., in press). Importantly, emerging research has begun to investigate individual, relationship, and infant characteristics that moderate the magnitude of post-birth changes in the relationship.

The *Vulnerability-Stress-Adaptation* (VSA) model can be used to understand the impact of life events like the transition to parenthood on relationship functioning. According to this model, couples will adapt better to stressful events to the extent that they have fewer vulnerabilities and more personal resources (Karney and Bradbury, 1995). In line with this model, we will argue that *personal happiness* is a psychological resource that affects how well couples adapt to the changes that occur across the transition to parenthood. Happiness has been found to increase adaptability and effective problem solving (Bryan et al., 1996; Fredrickson, 1998; Lyubomirsky et al., 2005). In particular, we will investigate whether personal happiness as a resource affects changes in relationship commitment. Relationship commitment is a multidimensional construct that entails psychological attachment to the relationship, a long-term orientation regarding the relationship, and the intention to persist in the relationship (Rusbult et al., 1998; Arriaga and Agnew, 2001). It is an important indicator of relationship quality and stability (Rusbult, 1983; Le et al., 2010; Stanley et al., 2010), and emerging research has uncovered individual variation in changes in commitment after childbirth (Doss et al., 2009; Kamp Dush et al., 2014; Ferriby et al., 2015).

Despite the importance of commitment in romantic relationships, only a few studies have examined changes in commitment across the transition to parenthood. Understanding whether and when the transition to parenthood changes parental relationship commitment is important because parental relationship quality and stability affects both the psychological and the physical development of children (e.g., Booth and Amato, 2001; Van Eldik et al., 2020). Gaining insight in factors that influence relationship quality and stability across the transition to parenthood informs new parents and the professionals working with them. The main question we aim to answer in the present study is who are the parents that experience changes in relationship commitment across the transition to parenthood? We will test the general hypothesis that parents with more personal happiness prior to pregnancy will experience less change in relationship commitment after childbirth than parents with less prenatal happiness. In addition, we will explore whether happiness also predicts changes in relationship commitment for childless couples.

### Commitment Across the Transition to Parenthood

How does the transition to parenthood affect relationship commitment? One prediction would be that commitment decreases after child-birth, in accordance to the often observed declines in relationship satisfaction and relationship functioning. Adapting to the transition and the increases in negative interactions between partners may erode positive aspects of the relationship, including commitment (Doss et al., 2009). A contrasting prediction is that commitment increases after first childbirth, because the presence of children raises the investments in the relationship and increases the costs of ending a relationship (e.g., Rusbult, 1983; Rusbult et al., 1998). This corresponds to the concept of *constraint commitment* (Stanley et al., 2010): Investments can act as a constraint to ending the relationship, because terminating the relationship becomes more costly economically, socially, personally, or psychologically than staying in the relationship. In line with this idea, commitment has been found to increase with the number of children (Sorokowski et al., 2017). Studies found that parents are less likely to divorce than childless couples (Waite and Lillard, 1991), and that a higher number of children is related to a lower divorce rate (Cherlin, 2010).

The few studies to date on changes in commitment across the transition to parenthood suggest that commitment on average decreases after childbirth (Doss et al., 2009; Kamp Dush et al., 2014; Ferriby et al., 2015), supporting the idea that the transition and the increase in negative interactions between partners negatively affect commitment (Doss et al., 2009). What these studies have in common is that they found a considerable amount of individual variation. Despite the negative average trend, some partners showed stable or increased commitment after childbirth. The *Vulnerability-Stress-Adaptation* (VSA; Karney and Bradbury, 1995) model offers a paradigm for predicting such variability in relationship change across the transition to parenthood (Kluwer, 2010) and is increasingly used as a framework to investigate individual differences in changes across the transition to parenthood (e.g., Doss et al., 2009; Trillingsgaard et al., 2014; Ter Kuile et al., 2017). According to the VSA model, personal enduring vulnerabilities can aggravate the impact of a stressful event on relationship functioning. Vulnerabilities can be practical, such as financial scarcity, or psychological, such as insecure attachment. Based on this model, couples can be expected to fare worse across the transition to parenthood to the extent that partners have more enduring vulnerabilities prior to childbirth that decrease their ability to adapt. Although the focus of the VSA model is on vulnerabilities, research has shown that having *resources* diminishes the impact of the transition to parenthood on the relationship (e.g., Ter Kuile et al., in press) and increases parents' adaptation to parenthood (Ter Kuile et al., 2017). In the current study, we investigate individual happiness as a psychological resource that increases couples' ability to adapt to first-time parenthood.

### Happiness as an Individual Psychological Resource

In their review, Lyubomirsky et al. (2005) present evidence that happiness predisposes people to look on the bright side and that it relates to superior coping during difficult times. For example, Lyubomirsky and Tucker (1998) showed that happy participants, as compared to unhappy participants, tended to think about life events more favorably and positively, by seeing humor and didactic value in adversity and by emphasizing recent improvement in their lives. Fredrickson (1998, 2001) has argued that positive emotions such as happiness have an adaptive purpose by helping to prepare for future challenges. Positive emotions lead to greater creativity, exploration, and social behavior, and thereby increase physical, social, intellectual, and psychological resources (Fredrickson, 2004). These durable resources can diminish the impact of negative events that occur later on, increasing adaptability and resilience (Fredrickson, 2001). Studies have indeed found that positive emotions such as happiness broaden the scope of attention (Basso et al., 1996), cognition (Isen, 2009), and action (Renninger, 1992). Happy adults as well as happy children have been found to be better able to learn new tasks and to show more effective problem solving (Bryan et al., 1996).

Based on the foregoing, happy individuals likely will be better in coping with changes and difficulties they encounter across the transition to parenthood than unhappy individuals. We expect that those with higher levels of happiness are better able to adapt to the transition to parenthood, and therefore the relationship likely suffers less, than those with lower levels of happiness, which translates into more stable vs. declining commitment levels across the transition to parenthood.

We are not aware of previous work showing evidence for the protective effects of personal happiness across the transition to parenthood, although there is evidence for associations between relationship quality (including commitment) and personal happiness (e.g., Demir, 2008). Also, a few studies have examined related constructs as predictors of the effect of childbirth on relationship outcomes, such as life satisfaction and depression. Life satisfaction predicted relationship satisfaction across the transition to parenthood in mothers (Dyrdal et al., 2011). A few studies have found that depressive symptoms across the transition to parenthood are a risk factor for greater decreases in relationship quality (Feeney et al., 2003; Whisman et al., 2011; Trillingsgaard et al., 2014).

Traditionally, research on the transition to parenthood literature largely focuses on risk factors (such as depression) and less on potential protective factors (such as happiness). The question is of course whether risk factors and protective factors are two sides of the same coin, and whether the focus on risk factors is warranted. There is some evidence to suggest that happiness and depression are not bipolar opposites (Rafaeli and Revelle, 2006). To explore this question, we included a pre-pregnancy measure of depressive symptoms in additional analyses to test whether this would predict changes in commitment across the transition to parenthood.

### The Present Research

The present work aims to investigate how relationship commitment changes across the transition to parenthood, and whether changes in commitment vary as a function of individual pre-pregnancy happiness. Based on earlier research, commitment is theorized to decrease on average, but less so for those with sufficient resources to adapt across the transition to parenthood. We hypothesize that pre-pregnancy happiness predicts changes in commitment across the transition to parenthood, such that more prenatal happiness is related to a greater increase or smaller decrease in commitment after childbirth. In this study, we included both partners, in contrast to many previous studies. Because of the interdependence between partners (Kashy and Kenny, 2000), it is important to not only examine how parents' relationship commitment is affected by their own happiness, but also by their partner's happiness. This may be especially important across the transition to parenthood, as the intensive caretaking required by infants can foster interdependence even more.

We will compare changes experienced by first-time parents to changes experienced by couples who did not become parents during the course of this study. By including a comparison group of childless couples, the mere passage of time can be ruled out as an alternative explanation for any differences found in changes in relationship commitment (Doss et al., 2009; Lawrence et al., 2010). It enables us to test for possible pre-existing differences between couples that do and couples that do not have children. It also allows us to explore whether happiness is a stronger predictor of differential trajectories for parents than for childless couples. If happiness is indeed a resource that increases partners' adaptation, the effect of happiness on commitment should be stronger among couples who are going through a major life transition than a comparison group of couples who are not.

By including pre-pregnancy measurements, we can rule out that effects are due to changes that may occur during pregnancy (Lawrence et al., 2010). We further include measurements beyond the first year after childbirth to study the longevity of the effects of the transition to parenthood on commitment. Finally, we will explore gender differences as prior research has shown that fathers' commitment was more vulnerable to change across the transition to parenthood than mothers' commitment (e.g., Doss et al., 2009; Kamp Dush et al., 2014; Ferriby et al., 2015).

## Method

### Participants and Procedure

We used data from the Marriage and Well-being Survey that were collected at 5 time points among 199 newlywed couples, as part of a larger study (Finkenauer et al., 2009). T1 took place in 2005 within 2 months of marriage, and there was ~1 year between subsequent time points. During the course of the study, the majority of couples had their first child. Because we wanted to include pre-pregnancy data, 12 couples who already had children or stepchildren at T1 were excluded. In addition, 23 couples became parents between T1 and T2. Because we cannot verify whether these couples were already pregnant at T1 or not, these couples were also excluded. The final sample therefore consisted of 109 couples (66.5%) who became parents during the course of this study at different time points, and a comparison group of 55 couples (33.5%) who did not have children during this time.

Participants were recruited via the municipalities in which they got married. Inclusion criteria were that this was the couple's first marriage, that couples had no children in this marriage or from previous relationships, and that partners were between 25 and 40 years old. Of all couples that fulfilled the criteria, 19% agreed to participate in the study. This response rate is similar to that in other studies recruiting participants from public records in the United States (e.g., Kurdek, 1991). At all data collections, both members of the couple separately filled out an extensive questionnaire at home in the presence of a trained interviewer. The questionnaire took about 90 min to complete. Partners were instructed not to discuss the questions or answers with each other. At each data collection, couples received 15 euro and a small gift (e.g., a book, a pen set) after they completed their questionnaires. All procedures were in compliance with the research and consent protocol of the Faculty of Social Sciences of the Free University at Amsterdam.

Of the participants, 128 (58.7%) became parents between T2 and T3, 58 (26.6%) between T3 and T4, and 32 between T4 and T5 (14.7%). The comparison group consisted of 110 participants (33.5%) who did not have children during the study. There was a relatively low attrition rate in this longitudinal study. At T2, 320 of the initial 328 couples still participated in the study, T3 consisted of 310 participants, T4 of 268, and T5 of 240 (73.2% of the sample at T1).

The mean age of husbands was 31.88 years (*SD* = 4.81) and the mean age of wives was 29.17 years (*SD* = 4.34) at T1. Couples had been romantically involved for 5.75 years (*SD* = 3.05) on average and had been living together for an average of 3.66 years (*SD* = 2.20) at T1. Nearly all couples had the Dutch nationality (97.6% of the husbands and 94.5% of the wives). Of the husbands, 18.3% was lower educated (high school or less), 18.9% completed community college (technical or vocational education), 29.9% had finished college (bachelor's degree), and 25.0% had finished university (master's degree). Of the wives, 12.7% was lower educated, 17.7% completed community college, 37.8% had finished college, and 24.4% had finished university. At T1, 98.2% of the husbands and 93.0% of the wives had a paid job. The modal number of working hours was 33 to 40 h a week (69.9% of the husbands and 50.6% of the wives). All the pregnancies were planned.

### Measures

#### Commitment

Commitment to the relationship was measured with 8 items, adapted from the investment model scale (Rusbult et al., 1998). The scale demonstrated good convergent and discriminant validity, and predicted later relationship quality and stability in prior studies (Rusbult et al., 1998). An example item is “*I hope that the bond that I have with my partner will stay the way it is now for a long time*.” Answers were rated on a 5-point scale (1 = *never*, 5 = *always*). Cronbach's alpha ranged between 0.87 and 0.90 for men and 0.90 and 0.93 for women across the 5 time points.

#### Happiness

Global subjective happiness was measured with a 4-item scale developed by Lyubomirsky and Lepper (1999). The scale was found to have a stable and good internal consistency across five different populations in 14 studies (Lyubomirsky and Lepper, 1999). An example item is “*In general, I consider myself:*” and “*Compared to most of my peers, I consider myself:*”. Participants rated their answer on a 7-point scale (1 = *not a very happy person*, 7 = *a very happy person*). Cronbach's alpha was 0.73 for men and 0.75 for women at T1.

#### Depression

Depression was measured using the Centre for Epidemiologic Studies Depression scale (CES-D; Radloff, 1977). The scale consists of 20 items that measure how often participants experienced depressed affect, positive affect (reverse coded), and somatic and retarded activity during the past week. Items were rated on a 4-point scale [1 “*Never or rarely (less than 1 day)*” to 5 “*Usually or always (5–7 days)*”]. The CES-D has been found to have a high internal consistency and validity in numerous studies (Eaton et al., 2004). Cronbach's alpha was 0.83 for men and 0.86 for women at T1.

#### Analyses

To test our predictions, we applied latent growth curve modeling. The intercept in the models corresponded with the average level of commitment at T1 (prior to pregnancy for the parents), and the linear slope represented the changes of commitment across time. Time since childbirth was included as a control variable in the parents' model. Both partners were included in the same model, in analogy to the principles of the Actor–Partner Interdependence Model (APIM: Kashy and Kenny, 2000).

To test our hypothesis that happiness predicts changes in commitment, we regressed the intercept and slope on the predictor happiness. The trajectories of parents and the comparison group of childless couples were analyzed in a multiple group dyadic growth model, allowing us to compare parents' and non-parents' trajectories. The models were first tested with all possible parameters included. The goal of an APIM analysis with distinguishable dyads is to test the fit of more parsimonious models that constrain estimates. Model fit that is not significantly worse after paths are constrained indicates that effects do not differ significantly (Peugh et al., 2013). Ideally, Chi-square is used to test whether changes in model fit are significant. Due to the complexity of this model however, Chi-square testing led to unstable results, depending on the order in which effects were constrained. We therefore placed constraints using model fit, assessed using the comparative fit index (CFI), Tucker Lewis index (TLI) and root mean square error of approximation (RMSEA). Acceptable model fit is generally defined as a cutoff value higher than 0.90 is for the CFI and lower than 0.08 for the RMSEA (Byrne and Crombie, 2003).

Models were estimated using version 7.4 of the statistical program M*plus* (Muthén and Muthén, 2018). Little (1988) missing-completely-at-random test showed that the pattern of missing data did not fully resemble a completely at random pattern [χ^2^ (187, *N* = 164) = 200.86, *p* = 0.03]. Inspection of this pattern showed it only to be a factor of time, such that attrition increased at each wave. Since this is inherent to longitudinal studies, and missingness was not related to any other main variable or demographics, we included all available data using a Full Information Maximum Likelihood procedure (which already provides good estimates with MAR). The output files of all the models are available upon request from the first author.

## Results

### Growth Model of Average Commitment and Change in Commitment Over Time

We first examined correlations between all the main variables at T1 (see Table 1). Happiness and commitment were moderately correlated for parents and childless men, but uncorrelated for childless women. Next, we examined the average intercept (I) at T1 and average slope (S) of commitment for both parents and childless men and women (see Table 2). As happiness was not yet included, this model shows the unconditional estimates of mean commitment at T1 and changes in commitment across time. In addition, the variances around these growth factors are estimated. The variance reflects the individual variation in average level or rate of change.

**Table 1:** Intercorrelations, means, and standard deviations of variables at T1.

**Variables**	**1**	**2**	**3**	**M (SD)**	**M (SD)**
				***Fathers***	***Mothers***
1. Happiness	0.21*	0.45^**^	−0.34^**^	5.80 (0.63)	5.86 (0.69)
2. Commitment	0.24^*^	**0.16**	−0.26^**^	4.66 (0.3*9*)	4.73 (0.33)
3. CESD	−0.53^**^	−0.08	**0.12**	1.30 (0.27)	1.34 (0.31)
				***Childless Men***	***Childless Women***
1. Happiness	**0.07**	0.02	−0.37^**^	5.66 (0.90)	5.73 (0.87)
2. Commitment	0.28^*^	**0.14**	0.06	4.64 (0.38)	4.76 (0.31)
3. CESD	−0.46^**^	0.01	**0.17**	1.33 (0.27)	1.45 (0.37)

* *p < 0.05*,

** *p < 0.01. Values for women are above the diagonal, values for men are below. Correlations between husbands and wives are presented in bold on the diagonal*.

**Table 2:** Means (intercepts) before pregnancy and changes (slopes) across time.

	**Intercept (mean level)**		**Slope (rate of change)**	
**Commitment**	**M (*SE*)**	**Variance (*SE*)**	**M (*SE*)**	**Variance (*SE*)**
Fathers	4.64 (0.03)	0.10 (0.02)^***^	−0.01 (0.02)	0.001 (0.002)
Mothers	4.73 (0.03)	0.08 (0.01)^***^	−0.03 (0.01)^**^	0.004 (0.002)^*^
Childless Men	4.64 (0.03)	0.10 (0.02)^***^	−0.03 (0.01)^**^	0.004 (0.002)^*^
Childless Women	4.73 (0.03)	0.08 (0.01)^***^	−0.03 (0.01)^**^	0.004 (0.002)^*^

* *p < 0.05*,

** *p = 0.001*,

*** *p <0.001*.

The final model had an acceptable fit, CFI = 0.956, TLI = 0.956, RMSEA = 0.054 (90% CI = 0.000, 0.083). Constraining the intercept (average level of commitment at T1) of fathers and childless men to be equal resulted in an increase model fit, indicating that their intercept did not differ significantly. Constraining the intercept of mothers and childless women similarly increased model fit. Constraining the intercept for men and women to be equal resulted in a decrease in model fit, suggesting their intercepts were not equal. Women reported higher levels of commitment than men at T1. Fathers and childless men had on average relatively high levels of commitment at the beginning of their marriage (*I* = 4.65*, p* < 0.001; variance = 0.10, *p* < 0.001). Mothers and childless women reported even higher initial levels of commitment (*I* = 4.73*, p* < 0.001; variance = 0.08, *p* < 0.001; see Table 2).

There was an increase in model fit when the slope was constrained to be equal for mothers, childless women and childless men as compared to the unconstrained model where all slopes were allowed to differ. This indicates that the slope of commitment (i.e., change over time) did not differ significantly between mothers, childless women, and childless men. Model fit decreased when fathers' slope was constrained to be equal, indicating that fathers' slope differed from mothers and childless men and women. Over time, both; mothers and childless partners experienced a slight but significant decline in commitment over time (*S* = −0.03*, p* = 0.001; variance = 0.004, *p* = 0.03). Fathers' slope was not significant (*S* = −0.01*, p* = 0.77; variance = 0.001, *p* = 0.47), indicating that their commitment did not change over time. Additional analyses with independent samples *t*-tests showed that there were no significant differences in average commitment between mothers and childless women, or between fathers and childless men, at any timepoint (analyses available upon request).

In sum, parents reported equally high levels of commitment at T1 as childless men and women, but mothers and childless women reported higher commitment at T1. Mothers and childless men and women experienced the same decline in commitment in the years after their marriage, while fathers' commitment remained stable.

### Commitment Predicted by Happiness

In the next step, happiness at T1 (prior to pregnancy) was included in the model as a predictor of the intercepts and slopes of commitment (see Table 3). The model includes both the effect of the individual's happiness on their own commitment (*actor effect*) as well as the effect on their partner's commitment (*partner effect*) of fathers and mothers and childless men and women. To test our hypothesis, we looked at the predictive effects of actor and partner happiness on changes in commitment across the transition to parenthood for parents and compared them to childless men and women (i.e., the effect of happiness on the slopes).

**Table 3:** Actor and partner effects of happiness on the intercept and slope of commitment.

	**Actor effects of happiness**						**Partner effects of happiness**					
**Commitment**	**Intercept**			**Slope**			**Intercept**			**Slope**		
	***b***	***SE***	**β**	***b***	***SE***	**β**	***b***	***SE***	**β**	***b***	***SE***	**β**
Fathers	0.13^***^	0.09	0.30	0.02^**^	0.01	0.43	−0.01	0.02	−0.03	0.02^**^	0.01	0.46
Mothers	0.25^***^	0.04	0.58	−0.01	0.01	−0.06	−0.01	0.02	−0.02	0.02^**^	0.01	0.19
Childless men	0.13^***^	0.04	0.32	−0.01	0.01	−0.17	−0.01	0.02	−0.03	−0.01	0.01	−0.17
Childless women	0.02	0.04	0.06	−0.01	0.01	−0.11	−0.01	0.02	−0.04	−0.01	0.01	−0.11

** *p < 0.01*,

*** *p < 0.001. b refers to the unstandardized coefficient, SE to the standard error of b, and β to the standardized coefficient*.

The original model had a poor fit (CFI = 0.946, TLI = 0.931, RMSEA = 0.065 (90% CI = 0.033, 0.091). The final model had an acceptable fit, CFI =0.964, TLI =0.961, RMSEA = 0.049 (90% CI = 0.000, 0.076). The actor effects of happiness on the intercepts showed that, as predicted, more reported happiness at T1 predicted higher average levels of commitment at T1 for fathers (unstandardized *b* = 0.13, *p* < 0.001; see Table 3 for standard error *SE* and standardized β). Model fit improved when this effect was constrained to be equal for childless men (*b* = 0.13, *p* < 0.001), indicating that the effect did not differ between fathers and childless men. The effect of happiness on initial commitment was slightly larger for mothers (*b* = 0.25, *p* < 0.001) and not significant for childless women (*b* = 0.02, *p* = 0.69). The partner effects on the intercepts were not significant, showing that the partner's happiness at T1 did not predict the average level of commitment at T1 for parents and childless men and women (*b* = 0.01, *SE* = 0.02, β = 0.06, *p* = 0.66).

The effects of happiness on the slopes showed that fathers' own pre-pregnancy happiness predicted their change in commitment over time. There were also partner effects: Mothers' pre-pregnancy happiness predicted fathers' slope and fathers' happiness predicted mothers' slope. Model fit increased when fathers' actor effect was constrained to be equal to these partner effects (*b* = 0.023, *p* = 0.007). There was no actor effect for mothers; in other words, mothers' happiness did not predict their own change in commitment over time. Neither did own or partner happiness predict the slope of childless men and women. Model fit improved when mothers' actor effect was constrained to be equal to the partner and actor effects of childless men and women (*b* = −0.01, *p* = 0.32).

As predicted, happiness at T1 positively predicted changes in commitment across the transition to parenthood. Fathers' happiness prior to pregnancy positively predicted changes in their own and their partner's commitment over time, and mothers' happiness also predicted changes in fathers' commitment after childbirth. Happiness did not predict changes in commitment for men and women who did not have children during this time.

**Figure 1:**
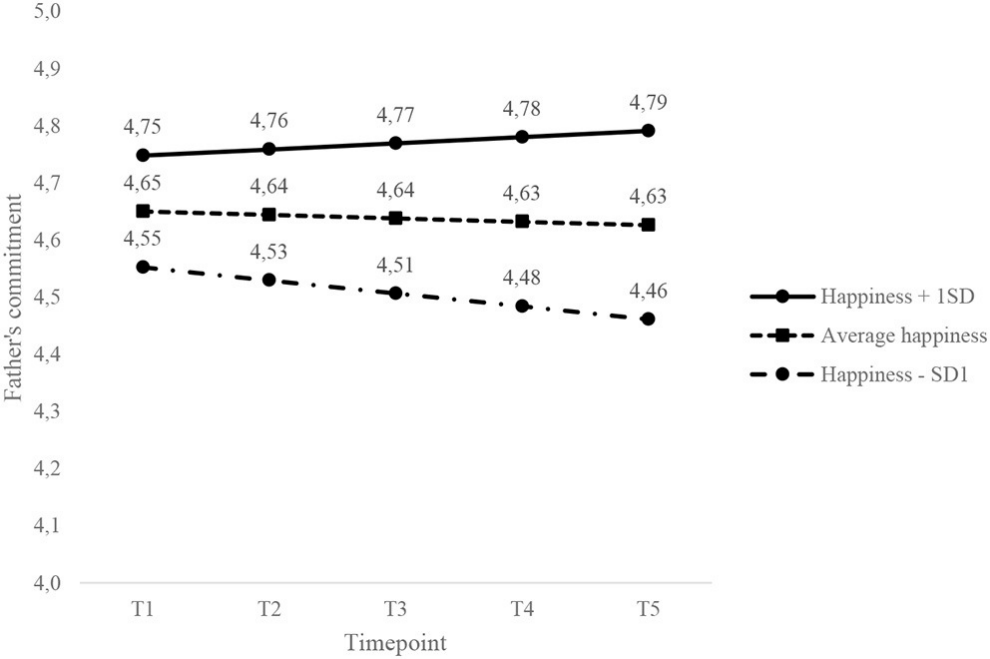
Effect of own happiness on the slope and intercept of fathers' commitment across the transition to parenthood. The y-axis is truncated to improve the visibility of the changes.

As shown in Figure 1, the commitment of happier fathers (i.e., +1 *SD* pre-pregnancy happiness) increased slightly in the years after childbirth. The commitment of fathers with average happiness prior to pregnancy remained stable, and the commitment of unhappier fathers (i.e., −1 *SD* pre-pregnancy happiness) decreased across the transition to parenthood. Because the effect of mothers' happiness on fathers' commitment was equal to fathers' actor effect, fathers with a happier partner at T1 showed the same increase as happier fathers, and fathers with an unhappier partner showed the same decrease as unhappier fathers (replicating Figure 1).

**Figure 2:**
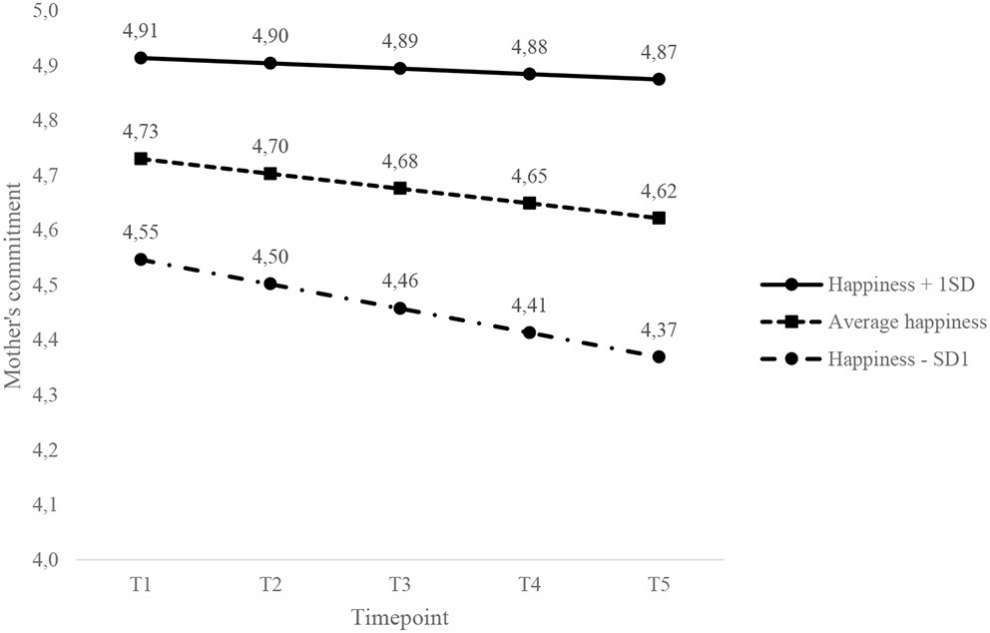
Effect of partner's happiness on the slope and intercept of mothers' commitment across the transition to parenthood. The y-axis is truncated to improve the visibility of the changes.

Figure 2 shows that the commitment of mothers with a happier partner prior to pregnancy decreased only slightly across the transition to parenthood and showed a steeper decline for mothers with a partner with average or lower happiness. The commitment of childless men and women decreased at the same rate, regardless of their own or their partner's T1 happiness.

### Commitment Predicted by Depression

An attempt at adding depression as covariate to the happiness model resulted in very poor model fit. Instead, an additional model was estimated with depression as a predictor, in order to indirectly compare its strength as a predictor of change in commitment over time to happiness. Model fit was acceptable [CFI = 0.977, TLI = 0.975, RMSEA = 0.038 (90% CI =0.000, 0.068)]. The results showed that there were no actor or partner effects of depressive symptoms on changes across time in commitment (see Table 4). Thus, depressive symptoms before pregnancy did not predict changes in commitment across time for either parents or childless men and women, in contrast to pre-pregnancy happiness as a predictor.

**Table 4:** Effects of depressive symptoms on the slope of commitment.

	**Actor effects of depression on the slope**			**Partner effects of depression on the slope**		
**Commitment**	***b***	***SE***	**β**	***b***	***SE***	**β**
Fathers	–	–	–	–	–	–
Mothers	–	–	–	−0.05	0.03	−0.17
Childless men	0.03	0.04	0.39	0.07	0.05	1.05
Childless women	0.03	0.04	0.19	−0.05	0.03	−0.23

*Where no effect is reported (–), model fit improved when the effect was constrained to 0, indicating effect did not significantly differ from 0*.

*b refers to the unstandardized coefficient, SE to the standard error of b, and β to the standardized coefficient*.

## Discussion

The current research extends previous work on relationship changes across the transition to parenthood in two important ways. First, we investigated changes in commitment, a largely unexplored factor despite it's crucial importance to relationship stability. Second, we studied the role of individual happiness as a psychological resource and argued that happy partners are better able to cope during difficult times, protecting them against a decrease in commitment across the transition to parenthood.

Mothers and childless men and women experienced a slight but significant decrease in commitment over the first 4 years of their marriage. Interestingly, fathers' commitment remained stable over time. This finding is consistent with findings that women's marital satisfaction declines to a greater extent than men's across the transition to parenthood (Twenge et al., 2003; Kluwer, 2010). Children can be viewed as an investment in the relationship (e.g., Rusbult et al., 1998) and terminating the relationship becomes more costly economically as well psychologically, thereby increasing *constraint commitment* (Stanley et al., 2010). In line with this, and contrary to findings on relationship satisfaction (Twenge et al., 2003), parents did not experience a stronger decrease in commitment than men and women who did not have a child during the course of this 4 year study. This could also be explained by the fact that relationship quality in general tends to decrease over time, regardless of parenthood. Average declines in relationship evaluations are evident across prior studies modeling trajectories of change (e.g., Lavner and Bradbury, 2010; Overall, 2018).

A main contribution of the current research is that we showed that changes in commitment varied as a function of parents' pre-pregnancy levels of happiness. As predicted, the level of happiness prior to pregnancy predicted changes in commitment over time among those who became parents. The commitment of happier fathers (i.e., +1 *SD* pre-pregnancy happiness) and fathers with a happier partner increased slightly in the years after childbirth, whereas the commitment of unhappier fathers (i.e., −1 *SD* pre-pregnancy happiness) and fathers with an unhappier partner decreased across the transition to parenthood. In addition, the commitment of mothers with a happier partner prior to pregnancy decreased only slightly across the transition to parenthood and showed a steeper decline for mothers with a partner with average or lower happiness. Also as hypothesized, personal happiness at the beginning of marriage was a predictor of changes over time in commitment for parents but not for men and women who remained childless. The effects of happiness therefore seem to be stronger in couples who experience a major life change than among those who remained childless.

Our results are in line with the broaden-and-build theory that positive emotions increase adaptability (Fredrickson, 2001). In addition, our findings extend the VSA model (Karney and Bradbury, 1995) that proposed that partners' vulnerabilities exacerbate the effect of stressful situations on the marital relationship, by showing that psychological resources can protect the relationship during a major relationship transition. We acknowledge that our results might only apply to the transition to parenthood, which although undeniably a time of many changes that are potentially stressful, is also experienced as a very positive event by most parents. However, it is also conceivable that successful adaptation to less positive events can lead to improvements in relationships. Relationships have for example been found to become stronger after successful adaptation to negative life events, such as cancer (Gritz et al., 1990). Further research is needed to see whether our findings generalize to less positive relationship transitions.

Additional analyses indicated that happiness was a better predictor of changes in commitment across the transition to parenthood than depressive symptoms, although this could only be compared indirectly. This is in line with the broaden-and-build theory that argues that the function of positive emotions is not the reverse equivalent of the function of negative emotions (Fredrickson, 1998). Positive emotions broaden an individuals' thought–action repertoires, thereby building their personal resources. The personal resources gained through positive emotions can last much longer than the emotional state that initially lead to the increase in positive emotions (Fredrickson, 1998). Our results are in line with this theory, showing that happiness even several years prior to pregnancy relates to changes in the quality of the relationship of parents going through the transition to parenthood, and that these effects are not reverse of the effects of pre-pregnancy depressive symptoms. A possible limitation is the low level of depressive symptoms in this sample, which might cause low correlations due to a floor effect. This probably does not fully explain the lack of impact on commitment however, as depression correlated moderate to strongly with happiness.

Surprisingly, mothers' change in commitment across the transition to parenthood was only predicted by their partner's happiness, but not by their own happiness prior to pregnancy. It is possible that happier fathers are more involved in child care. The wives of fathers who report higher paternal involvement in child care tend to be more satisfied with their relationship, leading to greater marital stability (Kalmijn, 1999). The effect of fathers' happiness on changes in mother's commitment may therefore reflect mothers' satisfaction with fathers' contribution to child care. Future research could explore paternal child care involvement as a mediator of changes in mothers' relationship quality across the transition to parenthood. In addition, future research should continue to explore and compare factors that predict how parental relationships fare across the transition to parenthood. The VSA model (Karney and Bradbury, 1995) suggests that many different factors can act as strengths or vulnerabilities for a couple, including both personal and situational characteristics. Which of these many possible factors has the greatest impact on how relationships fare across the transition to parenthood? Are personal characteristics stronger predictors of relationship quality after childbirth than situational factors? This would increase our understanding of how and when becoming parents has a negative or a positive impact on romantic relationships.

## Strengths and Limitations

This research makes an important contribution to the existing literature by focusing on explaining individual variability in relationship commitment across the transition to parenthood. The majority of studies on the transition to parenthood has found a negative impact of child-birth on the marital relationship (Twenge et al., 2003). As is being increasingly argued (e.g., Kluwer, 2010; Doss and Rhoades, 2017), studies that move beyond the study of average trajectories of change and focus on identifying important predictors of individual changes, can provide greater understanding of the underlying mechanisms of change across the transition to parenthood.

We used refined methodology to increase the strength of our conclusions. Firstly, we included pre-birth measurements of the predictors and outcome variable, allowing for a more reliable baseline than measures during pregnancy when many changes may have already taken place (Lawrence et al., 2010). Secondly, our inclusion of similar married couples who did not have children allowed for a comparison of relationship changes across the transition to parenthood to changes unrelated to childbirth. Lastly, the data included measurements up to 4 years after childbirth, enabling to study the stability of the changes that occurred after childbirth.

A methodological limitation is that due to the complexity of the model (a latent growth model with two groups, with a predictor) it was not possible to use Chi-square to test and compare effects. However, in most cases this limitation had little effect in our analyses because model fit often improved when a constraint was placed, indicating that the constraint is reasonable because the model is both more parsimonious as well as having better fit. When a constraint decreased model fit, we used the CFI, TLI and RMSEA to determine whether to keep a constraint or not. In this case, the decision was more subjective. Because of this limitation, future studies should replicate these findings with larger groups in order to make Chi-square testing possible. A replication with a larger control group is also necessary to confirm the differences we found between couples who became parents and childless couples. The differences found in this study may be due to the size of the control group being smaller than the parent group, limiting the power to find effects.

Another limitation is the relative homogeneity of our sample; all couples were married, all pregnancies were planned, and the majority of participants was highly educated. For example, the number of unmarried parents is quite high in the Netherlands (in 2015 four out of 10 Dutch children were born to unmarried women; Dutch Central Bureau of Statistics, 2016). This sample is therefore not completely representative of the Dutch population of new parents. We expect that a more diverse sample would show greater variation in changes in commitment across the transition to parenthood. This would limit ceiling effects, and could result in finding stronger effects. Perhaps because of this issue, the changes in commitment that parents experienced were relatively small, and the difference between happier and unhappier parents, although significant, were also small. Future research is needed to determine whether these differences are meaningful. For example, how do decreases in commitment develop over time beyond the fourth year of marriage? And do happier parents, whose commitment increases or remain stable, separate or divorce less often than unhappier parents who experience stronger decreases in commitment?

## Conclusion

The results suggest that happiness prior to pregnancy may play a protective role across the transition to parenthood, by increasing the adaptability of first-time parents. Unhappier fathers, fathers with unhappier partners, and mothers with unhappier partners appeared to become more vulnerable to decreases in commitment after childbirth, while the commitment of happier fathers, fathers with a happier partner and mothers with a happier partner showed stability or even increases in commitment. Changes in commitment across the transition to parenthood were a function of pre-pregnancy happiness levels. Happiness only predicted changes in commitment for couples who became parents, but not for couples who remained childless. These findings support the idea that happiness is a resource with an adaptive function, playing a role in relationships during major life transitions. In addition, the findings showed that changes in the relationship of parents across the transition to parenthood can be predicted long in advance, even prior to pregnancy. This suggests that prenatal detection of couples in need of support is possible.

## Data Availability Statement

There is no public use file available for the data used in the present study. Requests to access the present data should be directed to martin.brunner@uni-potsdam.de.

## Ethics Statement

Ethical review and approval was not required for the study on human participants in accordance with the local legislation and institutional requirements. The patients/participants provided their written informed consent to participate in this study.

## Author Contributions

HT, EK, and TL developed the research question. CF designed the study and collected the data. HT conducted the analyses and wrote the first draft of the paper. EK revised the manuscript. EK, TL, and CF provided feedback and approved the final version of the manuscript. All authors contributed to the article and approved the submitted version.

## Conflict of Interest

The authors declare that the research was conducted in the absence of any commercial or financial relationships that could be construed as a potential conflict of interest.
