# Supplementary material for: Changes in Relationship Commitment Across the Transition to Parenthood: Pre-pregnancy Happiness as a Protective Resource
Source: Front Psychol. 2021 Feb 16;12:622160. doi: 10.3389/fpsyg.2021.622160 (PMC7921486; doi:10.3389/fpsyg.2021.622160)
Supplement: Supplementary file 1 [file Data_Sheet_1.PDF]

Supplemental materials for:

Changes in Relationship Commitment Across the Transition to Parenthood:
Pre-pregnancy Happiness as a Protective Resource

Appendix 1: *Mplus* syntax *of the growth model of commitment*

Appendix 2: *Mplus Syntax of the model of the effect of happiness on commitment*

commit = male commitment
bcomm = female commitment
happin = male happiness
bhappi = female happiness
age_now = age of the child, i.e. time since childbirth

Appendix 1: *Mplus Syntax of the growth model of commitment*

```
  USEVARIABLES =
    bcomm_1 bcomm_2 bcomm_3 bcomm_4 bcomm_5 age_now
    commit_1 commit_2 commit_3 commit_4 commit_5;

    MISSING ARE ALL (999);
grouping is Parents( 0=control 1=parents);



ANALYSIS:
    MODEL = NOCOV;
    PROCESSORS = 4;
    ESTIMATOR = MLR;

MODEL:
    I by  commit_1 commit_2 commit_3 commit_4 commit_5;
    S by  commit_1 commit_2 commit_3 commit_4 commit_5;
    FI by  bcomm_1 bcomm_2 bcomm_3 bcomm_4 bcomm_5;
    FS by  bcomm_1 bcomm_2 bcomm_3 bcomm_4 bcomm_5;

     S on age_now;
    FS on age_now;

    [bcomm_1@0];
    [bcomm_2@0];
    [bcomm_3@0];
    [bcomm_4@0];
    [bcomm_5@0];

    [commit_1@0];
    [commit_2@0];
    [commit_3@0];
    [commit_4@0];
    [commit_5@0];

   !----------------------------------

 model control:
    I BY commit_1@1 commit_2@1  commit_3@1 commit_4@1 commit_5@1;
     [I] (m10);
      I  (v10);
    S BY commit_1@0 commit_2@1  commit_3@2 commit_4@3 commit_5@4;
     [S] (m2);
      S  (v2);
```

```
 FI BY bcomm_1@1 bcomm_2@1 bcomm_3@1  bcomm_4@1 bcomm_5@1;
  [FI] (m1);
  FI  (v1);
 FS BY bcomm_1@0 bcomm_2@1 bcomm_3@2  bcomm_4@3 bcomm_5@4;
 [FS] (m2);
  FS  (v2);

I with S@0 (L1);
FI with FS@0 (L10);

I with FS (L2);
FI with S (L20);

I with FI (L30);
S with FS@0 (L40);

 S on age_now@0;
 FS on age_now@0;

 commit_1 with bcomm_1 (p1);
 commit_2 with bcomm_2@0 (p2);
 commit_3 with bcomm_3 (p3);
 commit_4 with bcomm_4 (p3);
 commit_5 with bcomm_5@0 (p5);

!-----------------------------------

 model parents:
  I BY commit_1@1 commit_2@1  commit_3@1 commit_4@1 commit_5@1;
  [I] (m10);
   I  (v10);
 S BY commit_1@0 commit_2@1  commit_3@2 commit_4@3 commit_5@4;
  [S] (m40);
   S  (v40);
  FI BY bcomm_1@1 bcomm_2@1  bcomm_3@1 bcomm_4@1  bcomm_5@1;
  [FI] (m1);
   FI  (v1);
  FS BY bcomm_1@0 bcomm_2@1  bcomm_3@2 bcomm_4@3  bcomm_5@4;
  [FS] (m2);
   FS  (v2);
!   FS@0;

I with S (L5);
FI with FS@0 (L50);

I with FS (L6);
FI with S (L60);
```

```
  I with FI@0 (L3);
  S with FS (L4);

   S on age_now;
   FS on age_now@0;

  commit_1 with bcomm_1 (p6);
   commit_2 with bcomm_2 (p6);
   commit_3 with bcomm_3@0 (p8);
   commit_4 with bcomm_4 (p6);
   commit_5 with bcomm_5 (p10);


OUTPUT: TECH1 TECH4 SAMPSTAT STANDARDIZED modindices (4) cinterval;
```

Appendix 2: *Mplus Syntax of the model of the effect of happiness on commitment*

```
  USEVARIABLES =
    bcomm_1 bcomm_2 bcomm_3 bcomm_4 bcomm_5 age_now
    commit_1 commit_2 commit_3 commit_4 commit_5 happin_1 bhappi_1;

    MISSING ARE ALL (999);
grouping is Parents( 0=control 1=parents);



ANALYSIS:
    MODEL = NOCOV;
    PROCESSORS = 4;

MODEL:
    I by  commit_1 commit_2 commit_3 commit_4 commit_5;
    S by  commit_1 commit_2 commit_3 commit_4 commit_5;
    FI by  bcomm_1 bcomm_2 bcomm_3 bcomm_4 bcomm_5;
    FS by  bcomm_1 bcomm_2 bcomm_3 bcomm_4 bcomm_5;

    [bcomm_1@0];
    [bcomm_2@0];
    [bcomm_3@0];
    [bcomm_4@0];
    [bcomm_5@0];

    [commit_1@0];
    [commit_2@0];
    [commit_3@0];
    [commit_4@0];
    [commit_5@0];

    I on happin_1;
    FI on bhappi_1;
    S on happin_1;
    FS on bhappi_1;

    FI on happin_1;
    I on bhappi_1;
    FS on happin_1;
    S on bhappi_1;

    happin_1 with bhappi_1;

    S on age_now;
    FS on age_now;
```

```
 !-----------------------------------
   model control:
    I BY commit_1@1 commit_2@1  commit_3@1 commit_4@1 commit_5@1;
     [I] (m10);
      I  (v10);
    S BY commit_1@0 commit_2@1  commit_3@2 commit_4@3 commit_5@4;
     [S] (m20);
      S  (v20);
    FI BY bcomm_1@1 bcomm_2@1 bcomm_3@1  bcomm_4@1 bcomm_5@1;
     [FI] (m1);
     FI  (v1);
    FS BY bcomm_1@0 bcomm_2@1 bcomm_3@2  bcomm_4@3 bcomm_5@4;
    [FS] (m2);
     FS  (v2);

   I with S@0 (L1);
   FI with FS@0 (L10);

   I with FS (L2);
   FI with S (L20);

   I with FI (L30);
   S with FS@0 (L40);

   happin_1 with bhappi_1(h1);

    I on happin_1(cor1);
    FI on bhappi_1(cor77);
    S on happin_1(cor20);
    FS on bhappi_1(cor20);

    FI on happin_1 (cor7);
    I on bhappi_1(cor7);
    FS on happin_1 (cor20);
    S on bhappi_1 (cor20);

    S on age_now@0;
    FS on age_now@0;

    commit_1 with bcomm_1 (p1);
    commit_2 with bcomm_2@0 (p2);
    commit_3 with bcomm_3 (p3);
    commit_4 with bcomm_4 (p4);
    commit_5 with bcomm_5@0 (p5);

!-----------------------------------
    model parents:
```

```
  I BY commit_1@1 commit_2@1  commit_3@1 commit_4@1 commit_5@1;
  [I] (m30);
   I  (v30);
 S BY commit_1@0 commit_2@1  commit_3@2 commit_4@3 commit_5@4;
  [S] (m40);
   S  (v40);
  FI BY bcomm_1@1 bcomm_2@1  bcomm_3@1 bcomm_4@1  bcomm_5@1;
  [FI] (m3);
   FI  (v3);
  FS BY bcomm_1@0 bcomm_2@1  bcomm_3@2 bcomm_4@3  bcomm_5@4;
  [FS] (m4);
   FS  (v4);

I with S (L5);
FI with FS@0 (L50);

I with FS (L6);
FI with S (L60);

I with FI@0 (L3);
S with FS (L4);

 happin_1 with bhappi_1(h10);

 I on happin_1(cor1);
 FI on bhappi_1(cor50);
 S on happin_1(cor8);
 FS on bhappi_1(cor20);

 FI on happin_1 (cor7);
 I on bhappi_1(cor7);
 FS on happin_1 (cor8);
 S on bhappi_1 (cor8);

 S on age_now;
 FS on age_now@0;

 commit_1 with bcomm_1 (p10);
 commit_2 with bcomm_2 (p20);
 commit_3 with bcomm_3@0 (p30);
 commit_4 with bcomm_4 (p40);
 commit_5 with bcomm_5 (p50);

OUTPUT: TECH1 TECH4 SAMPSTAT STANDARDIZED modindices (4) cinterval;
```
